# Children and Adolescent’s Perception of Media Device Use Consequences

**DOI:** 10.3390/ijerph18063048

**Published:** 2021-03-16

**Authors:** Giulia Spina, Elena Bozzola, Pietro Ferrara, Nicola Zamperini, Francesco Marino, Cinthia Caruso, Livia Antilici, Alberto Villani

**Affiliations:** The Italian Pediatric Society, via Gioberti 60, 00165 Rome, Italy; giulia.spina@opbg.net (G.S.); pietro.ferrara@unicatt.it (P.F.); nzamperini@gmail.com (N.Z.); francesco.marino@opbg.net (F.M.); ufficiostampasip@gmail.com (C.C.); livia.antilici@opbg.net (L.A.); alberto.villani@opbg.net (A.V.)

**Keywords:** social media, children, adolescents, social network

## Abstract

Media device (MD) use is increasing worldwide among children. Adolescents and young children spend a lot of time using MD, Internet, and social networks. The age of initial use is getting lower to 12 years old. The aim of this research is to study children’s use and perception of MD. The Italian Paediatric Society (SIP) conducted a Survey on Italian children in collaboration with Skuola.net using an online questionnaire. A total of 10,000 questionnaires were completed. Children admitted they spend more than 3 h (41%), more than 2 h (29%), more than 1 h (21%) and less than 1 h (9%) daily. Problematic MD use has been found with children using MD before sleeping (38%), during school (24%), and at wake up in the morning (21%). Addiction was documented in 14% of adolescents. Among the reported consequences, low academic outcomes, and reduced concentration (24%), neck and back pain (12%), insomnia (10%), and mood disturbances (7%) were referred. Adolescents may have a low perception of the risks related to excessive MD. The duration of time spent using media devices is a main risk factor. In this context, parents should strongly discourage excessive MD use, mainly during school, at bedtime, and wake-up. Additionally, parents should be informed and start conversations with their children on the potential negative effects of prolonged MD use.

## 1. Introduction

Media device (MD) use is exponentially spreading among children and adolescents worldwide: in Italy, an ISTAT analysis reported that 85.8% of Italian adolescents aged 11–17 years regularly use smartphones, and over 72% access Internet via smartphones [1]. The majority of children in 19 European countries report using their smartphones "daily" or "almost all the time", according to the report EU Kids Online 2020: Survey results from 19 countries by the London School of Economics. In 11 countries (Croatia, Czech Republic, Germany, Estonia, Italy, Lithuania, Norway, Poland, Portugal, Romania, and Serbia), over 80% of children aged 9–16 use a smartphone to access the Internet at least once a day. In 2010, the number of children going online from their mobile phones ranged from 31% (Norway) to only 2% (Romania) [2]. Nearly all U.S. teens (95%) say they have access to a smartphone and 45% they are “almost constantly” on the Internet [3].

Adolescents and young children spend a lot of time with their smartphones browsing social media, especially Facebook, Instagram, and Twitter [4]. In particular, the age of initial use of social network is getting lower to 12–13 years probably reflecting the need to create a personal social identity [5]. Smartphones are quickly becoming the preferred MD for younger due to their screen size, interactive ability, capability of streaming content such as videogames, videos, photos, and applications [6]. Moreover, many children have their own MD which facilitate a prolonged and frequent use [7]. 

A growing body of literature links a prolonged use of digital media with psychosocial and physical negative consequences. In fact, negative and positive consequences of excessive smartphone use have been described in several studies [8,9,10]: reduction in concentration and academic performances, sleep disorders, ocular disturbances, obesity, addiction, cyberbullying but also improvement of self-control and emotional support [8,9]. Nevertheless, adolescents may have a low perception of the risks related to an excessive MD [10]. Few studies explored the perception of risk by adolescents and, among them, a European study reported that children are aware that MD may cause “lack of sleep, problem in doing homework and with study concentration” [11,12]. In this scenario, parents and caregivers have a crucial role in monitoring children and giving them the optimal behaviour for MD use [6]. 

The aim of this study is to investigate Italian children’s attitude toward MD, exploring their relation with technology (type of MD used, time spent using MD, and activities performed with MD) and their awareness about either MD correct use or health consequences. The results may be useful to reinforce messages on MD risks in children, helping clinicians, parents, and caregivers to face with children’s MD use. 

## 2. Material and Methods

On the base of the perception of the increased use of MD among children, as reported by previous studies, the Italian Paediatric Society (SIP) Scientific commission planned a qualitative research [13]. In details, it elaborated and conducted an anonymous and volunteer survey on MD use to Italian children and adolescents through social channels. The survey had been conducted in collaboration with a digital platform, “skuola.net”, which is provided of an official web site (Available from www.skuola.net, accessed 1 December, 2020) to which children may sign in with the authorization of their parents. As a consequence, another authorization is not required to enter the survey. The Ethic Approval was not required as the questionnaire was anonymous and in no way is possible to link the answer to the responder. In fact, the study is non-intrusive and does not involve direct interaction between the researcher and individuals through the Internet. Finally, there is no expectation of privacy [14]. Questions have been uploaded as an appendix. The study period of the descriptive qualitative study ranged from 1 September 2019 to 1 November 2019, before the COVID-19 pandemic outbreak. In details, research questions of the study are as following:

1. What is the relationship between children and MD?
2. Which is children perception about MD risks and opportunities?
3. Which is parents’ attitude with children’s MD usage?

The online questionnaire used was adapted from the European Union Kids Online Survey 2010 [12]. It was available on the digital platform of skuola.net, on SIP website as well as on the social network pages (Twitter, Facebook, LinkedIn, and Telegram). The unique inclusion criteria were aged 9–18 years. The inclusion criteria were well specified in both the digital platforms and social network pages. Data processing was performed with SPSS 26 software, Released 2019, IBM SPSS Statistic for Windows, Version 26.0 Armonk, NY, USA: IBM corp and comparison of categorical variables was done by the χ^2^ test and goodness-of-fit test.

## 3. Results

A total of 10,000 online questionnaires was completed in the study period. Out of the sample size, 61% were male and 39% were female. According to our results, 87% of children younger than 18 years old has his own smartphone and 59% his own tablet. Just 2% and 4% of the responders use parents’ smartphone and tablet, respectively. In particular, smartphone owner has been more documented in patients older than 15 years old while tablet usage was described especially in patients younger than 15 years old (*p* < 0.001). In most cases, the first MD to be received was a smartphone (56%). As for the others, 27% got firstly a tablet and 17% a computer. Moreover, a percentage of 24% of children younger than 15 years old declared they received their first MD before 9 years old compared with 13% in children older than 15 years old (*p* < 0.001). 

Table 1 summarizes the results.

The first research question of our study was to understand children relationship with MD. Considering time of usage, the interviewed students generally spend a long time on MD: 41% more than 3 h, 29% more than 2 h, and 21% more than 1 h daily. Just 10% referred a MD use less than 1 h each day. Adolescents aged 15–18 years old were more likely to report a MD usage longer than 3 h daily (44%) compared to 9–14 years old participants with a percentage of 32% (*p* < 0.001). Problematic media use (PMU) has been reported by the responders. In particular, children younger than 15 years old were found to be more unable to renounce to MD compared with older children (*p* < 0.001). The main reason for smartphone use was the socio-recreational one (73%). Adolescents admit they use it to communicate with friends (24%), to join social networks (23%), to watch videos or movies (18%), or to play videogames (8%). Just 19% responders use smartphone for school research and 8% to talk to parents. 

Second, we focused on children perception about MD risks and opportunities. Out of responders, 23% is aware to have a “negative” relationship with technology: the use of MD is only wasting time (10%) or the Internet use is wasting time (23%). Older adolescents are aware to have a negative relationship with MD compared to younger ones (*p* < 0.001). Moreover, even among those who declare to have a positive relationship with technology, 47% of adolescent claims, “I sometimes spend too much time browsing Internet”. As for activities, 38% of the interviewed use smartphones before sleeping, 24% during school lessons and 21% at arousal. Moreover, 5% of the responders sleep with the MD under the pillow. 

According to our results, the interviewed are scarcely aware of negative consequences, as 26% referred no problems linked to a prolonged MD usage. As for the others, a reduced concentration during school activities have been referred by 24% of the participants, neck and back pain by 12%, insomnia, and mood disturbances by 10% and 7%, respectively. In most cases, adolescents are not aware how to improve a positive relationship with technology, not using MD during homework (33%), during conversation with friends or family (21%), before sleeping (19%), or during meals (16%).

Finally, we investigated parents’ attitude with children’s MD usage. Considering habits, parents seems to be aware of negative consequences related to MD usage giving rules and reprimanding children in a percentage of 42% and 37% respectively. More details are presented in Table 2 and Table 3. 

## 4. Discussion and Conclusions

Rapid technology developing facilitate MD use as people may connect almost at any time and in any place through mobile devices. As a consequence, the major concern is an excessive screen time use, over the recommended 2 h per day limit [12]. As technology becomes in most cases a larger part of children and adolescents’ daily activities, the risk of sequelae on physical, psychological, social, and neurological development are areas of concern. The technological progress and the emergence of multi-function devices have increased the perceived need to be always connected to multiple MD [15]. As a consequence, people consult MD before sleeping or at arousal and are unwilling to separate from it. In our sample size, most of the interviewed declared to consult MD at bedtime, early morning or even at school. Moreover, they are not able to renounce to the MD, so that they even sleep with it under the pillow (5%). Nevertheless, adolescents may have a low perception of the risks related to an excessive MD, in particular of addiction. MD addiction may be compared to substance-use addiction, so that it can be investigated through the awareness of adolescents to separate from technology [9]. Out of the responders, 15% is aware to be unable to separate from the own MD. Concern has been rising because Internet addiction is increasing among the youngest [16], as well as in literature where a higher percentage of responders cannot separate from the MD among the adolescents 9–14 years (18% vs. 13%, *p* < 0.001).

Apart from addiction, a growing body of literature links a prolonged use of digital media to psychosocial and physical negative consequences [17]. Excessive MD usage has been related to health problems involving sleep, learning, sight, mood, and muscles [9]. In some cases, children are aware of the negative consequences correlated to media use. In fact, European children declared that MD may cause “lack of sleep, problems in doing homework and with study concentration” [11]. 

The duration of devices consulting is one of the most important key components determining screen time effects, such as impaired vision and reduced daily concentration [10]. In our sample size as well, most of the negative consequences were reported among interviewed with a more than 3 h daily MD use. Out of our sample size, 38% referred the use at bedtime and 10% reported insomnia. Inadequate sleep, both in duration and quality may compromise physical and psychosocial developing in youth, as well as negatively affecting school performance [9,17]. Evening and night time exposure to bright light and blue light emitted by self-luminous devices may interfere with melatonin production, with an increased risk of sleep disturbance. Bedtime access to MD is associated with compromised sleep, insomnia and night arise [17,18]. 

Comparing our recent data to previous Italian report we observed an increment of children MD usage in the latest ten years. In particular, we noted an increased percentage of children using MD for more than 3 h (41% in 2019 versus 15.3% in 2010). As well, the percentage of children using MD for less than one hour is lower (9% in 2019 versus 21.4% observed in 2010). Details are presented in Table 4 [13].

Often children have their own personal device, which facilitate a more frequent use than in the previous years. In particular, a great increase of MD owner had been noted after the introduction of smartphones and after with iPhone in 2007, independently of family income. Indeed, 51% of children from lower-income households has their own smartphone [19]. A higher frequency of smartphone use and of its daily use has been found related to a problematic usage [20,21]. 

In this context, parents should strongly discourage the excessive use of MD, mainly during school, at bedtime, and wake-up. On the contrary, parents are encouraged to explain the potential negative effects of MD prolonged use and should start conversations to establish some rules. Our data revealed that a percentage of 20% of parents give a MD to their children when they are younger than nine years old, in line with other reports [22,23,24]. At this age, children are often immature and should be advised on potential risk while parents regularly monitor the daily use to prevent children MD addiction. 

The percentage of MD users, especially among minors, is growing, and so is the body of literature hinting at increasing rates of problematic smartphone use in children and adolescents. One of the most important aspect that need to be stressed is the perception by minors of excessive/problematic MD users. Improving children and adolescents’ awareness on MD use and their relationship with the technology is the first step to solve the problem. Previous studies demonstrated that self-reflection may increase the awareness of problems and seeds of change. By the way, a report on the relationship between children and mobile phone, pointed out that over 80% of the participants reported an increased awareness of their relationship dynamics with their child/parent, their own behaviour, or their communication styles after the survey. [25] In our questionnaire, 19% of responders use MD for school research and 8% for parents’ communications. As for the others, 24% use MD to communicate with friends, 23% to join social network, and 8% to play video games. A prolonged smartphone use for social networking or online chats may represent a risk factor for problematic usage [20,26]. In addition, gaming and stronger denial of game overuse were also found to predict smartphone addiction [20,21,26]. It is important to educate children before they approach to social network, managing the safety and security of their information. They should be instructed on the risk related to share personal information, get in contact with virtual friends and be engaged in dangerous contests or to face age-inappropriate contents. Moreover, the prolonged and excessive use of "electronic games" may affect the behaviour, dehumanization of the player, increasing the risk of depression and anxiety in frequent users. This study has been performed before the COVID-19 pandemic, which has been one of the greatest disruptions for everybody’s everyday lives, all around the world. One of the hardest involved categories has been, since the beginnings of the pandemic, children and adolescents, who suffered from the near-universal closing of schools. All of a sudden, most students almost everywhere in the world have found themselves experimenting remote learning, a completely new form of learning [27]. Some articles have been trying to understand what the effects of this kind of transition might have been for children and adolescents. For now, the main focus has been on digital divide, with a particular interest on the penalty for vulnerable and struggling students, while the high-achieving ones would have been minimally affected by the transition to the online world [28]. More studies are required to evaluate the impact of remote learning on MD use. 

A limit of the study is that the survey is based on self-reports provided by children and adolescents and so we cannot guarantee the honesty in survey responses. Nevertheless, 10,000 students, either with digital experience or without MD, entered the survey, or may be representative of students. As the volunteer questionnaire is anonymous, we can consider the results reliable and trustable. In fact, studies suggest that when participants on survey respond anonymously instead of confidentially, the disclosure of sensitive information is enhanced [29]. Further studies are required to confirm the data and the generalizability of the results. Other limitations of the study concern the socioeconomic status as well as school and family environments of the responders, which had not been investigated. In literature, socioeconomic medium and disadvantaged children spent, in average, more time using media devices compared with children from higher status. In addition, parents from more educated backgrounds set limits when children use screen devices, while lower parental education is associated with lower parental modelling [30].

In conclusion, in the last decade, a wide use of MD and technology has spread among children becoming an integral part of their life. Not all the youth may easily perceive negative the risks related to MD. Therefore, it is important to monitor adolescents’ MD usage and identify risk factors or inappropriate habits to support reflection on behaviour changes and communication styles, prompt strategies and seed goals. In this context, enhancing parents’ education and children communication may be useful to promote digital awareness and prevent adverse events connected to technologies.

## Figures and Tables

**Table 1:** Questionnaire about media device (MD) usage and perception among children and adolescents.

	%	Number	9–14 Years	Number	15–18 Years	Number
**Are you male or female?**		10,000		4000		6000
Male	61%	6100	55%	2200	70%	4200
Female	39%	3900	45%	1800	30%	1800
**Do you own a smartphone?**						
Yes	87%	8700	80%	3200	92%	5520
No	10%	1000	16%	640	7%	420
I use my parent’s smartphone	2%	200	3%	120	1%	60
I use my friend’s smartphone	1%	100	1%	40	0%	0
**Do you own a tablet?**						
Yes	59%	5900	63%	2520	57%	3420
No	36%	3600	32%	1280	38%	2280
I use my parent’s tablet	4%	400	4%	160	5%	300
I use my friend’s tablet	1%	100	1%	40	0%	0
**When did you receive your first electronic device?**						
Older than 9 years old	79%	7900	70%	2800	84%	5040
Younger than 9 years old	17%	1700	24%	960	13%	780
No answer	4%	400	6%	240	3%	180
**Which was your first electronic device?**						
Smartphone	56%	5600	48%	1920	59%	3540
Computer	17%	1700	13%	520	20%	1200
Tablet	27%	2700	39%	1560	21%	1260

**Table 2:** Questionnaire about MD usage and perception among children and adolescents.

	%	Number	9–14 Years	Number	15–18 Years	Number
**How many hours a day do you spend using an electronic device?**		10,000		4000		6000
<1 h	10%	1000	18%	720	7%	420
1–2 h	20%	2000	25%	1000	18%	1080
2–3 h	29%	2900	25%	1000	31%	1860
>3 h	41%	4100	32%	1280	44%	2640
**Are you able to give up on your smartphone or tablet?**						
For a day	16%	1600	16%	640	18%	1080
For three days	13%	1300	11%	440	14%	840
For a week	26%	2600	24%	960	26%	1560
For a month	30%	3000	31%	1240	29%	1740
Never	15%	1500	18%	720	13%	780
**How would you describe your relationship with technology?**						
Positive, but I sometimes spend too much time browsing Internet without realizing it	47%	4700	43%	1720	49%	2940
Positive, I use technology but without exceeding	30%	3000	36%	1440	27%	1620
Negative, I use my devices only wasting time	10%	1000	10%	400	9%	540
Negative, I use Internet wasting too much time online	13%	1300	11%	440	15%	900

**Table 3:** Questionnaire about MD usage and perception among children and adolescents.

	% 9–18 Years	Number
**For which activities do you mainly use your smartphone?**		10,000
Communicate with friends	24%	2400
Use social networks	23%	2300
School researches	19%	1900
Watch videos or movies	18%	1800
Play videogames	8%	800
Talking with parents	8%	800
**When you are at home, in what situations do you use your smartphone or tablet?**		
Before going to bed	38%	3800
While I do homework	24%	2400
As soon as I wake up	21%	2100
During meals	12%	1200
I always keep it under my pillow	5%	500
**When I use my smartphone or tablet, my parents…**		
They give me rules and times of use	42%	4200
They reprimand me	37%	3700
They watch contents with me	21%	2100
**Can you feel something different after you’ve used smartphones more than usual?**		
Lack of concentration during school activities	24%	2400
Eyes burning	21%	2100
Neck and back pain	12%	1200
I can’t sleep	10%	1000
I feel nervous	7%	700
None of these symptoms	26%	2600
**Which habits do you think you should improve to develop a positive relationship with technology?**		
Do not use it during homework	33%	3300
Do not use during conversations with friends or family	21%	2100
Do not use it before sleeping	19%	1900
Do not use it during meals	16%	1600
None of these	11%	1100

**Table 4:** Comparison of hours of MD usage in 2010 and 2019 in Italian.

Children		
Time (Hours)	2010 (%)	2019 (%)
<1 h	21.4	9
1–3 h	62.9	50
>3 h	15.3	41

## Data Availability

Availability of data and material at Bambino Gesù Children Hospital, Bozzola’s repository.

## Abbreviations

MD	media device
SIP	Italian Pediatric Society
